# Two classes of protective antibodies against Pseudorabies virus variant glycoprotein B: Implications for vaccine design

**DOI:** 10.1371/journal.ppat.1006777

**Published:** 2017-12-20

**Authors:** Xiangdong Li, Fanli Yang, Xule Hu, Feifei Tan, Jianxun Qi, Ruchao Peng, Min Wang, Yan Chai, Liying Hao, Junhua Deng, Chenyu Bai, Juan Wang, Hao Song, Shuguang Tan, Guangwen Lu, George F. Gao, Yi Shi, Kegong Tian

**Affiliations:** 1 National Research Center for Veterinary Medicine, High-Tech District, Luoyang, Henan, China; 2 CAS Key Laboratory of Pathogenic Microbiology and Immunology, Institute of Microbiology, Chinese Academy of Sciences, Beijing, China; 3 University of Chinese Academy of Sciences, Beijing, China; 4 West China Hospital Emergency Department, State Key Laboratory of Biotherapy, West China Hospital, Sichuan University, and Collaborative Innovation Center of Biotherapy, Chengdu, Sichuan, China; 5 College of Animal Science and Veterinary Medicine, Henan Agricultural University, Zhengzhou, Henan, China; University of Tübingen, GERMANY

## Abstract

Pseudorabies virus (PRV) belongs to the *Herpesviridae* family, and is an important veterinary pathogen. Highly pathogenic PRV variants have caused severe epidemics in China since 2011, causing huge economic losses. To tackle the epidemics, we identified a panel of mouse monoclonal antibodies (mAbs) against PRV glycoprotein B (gB) that effectively block PRV infection. Among these 15 mAbs, fourteen of them block PRV entry in a complement-dependent manner. The remaining one, 1H1 mAb, however can directly neutralize the virus independent of complement and displays broad-spectrum neutralizing activities. We further determined the crystal structure of PRV gB and mapped the epitopes of these antibodies on the structure. Interestingly, all the complement-dependent neutralizing antibodies bind gB at the crown region (domain IV). In contrast, the epitope of 1H1 mAb is located at the bottom of domain I, which includes the fusion loops, indicating 1H1 mAb might neutralize the virus by interfering with the membrane fusion process. Our studies demonstrate that gB contains multiple B-cell epitopes in its crown and base regions and that antibodies targeting different epitopes block virus infection through different mechanisms. These findings would provide important clues for antiviral drug design and vaccine development.

## Author summary

Pseudorabies virus (PRV) is an emerging veterinary pathogen that infects many domestic animals. Since 2011, highly pathogenic PRV variants have emerged in many farms in China and posed great economic burdens to the animal industry. However, the current marketed vaccines cannot provide effective protection against these emerging strains. The envelope glycoprotein B (gB) is a major viral antigen known to play a crucial role in PRV entry. In order to control PRV epidemics and treat associated diseases, we combined structural and immunological approaches to generate potential neutralizing antibodies targeting PRV gB and investigate their working mechanisms. A total of 15 monoclonal antibodies (mAbs) were identified with good neutralizing activity. Among them, fourteen mAbs target the domain IV of PRV gB and block virus entry though complement effect. In contrast, the remaining 1H1 mAb recognizes domain I of PRV gB, which can neutralize virus entry independent of complement and probably by interfering with the membrane fusion process. Our work reveals the structural details and immunogenic properties of PRV gB and may offer important guidance for developing antiviral therapeutics and vaccines against PRV infections.

## Introduction

Pseudorabies virus (PRV) belongs to the family *Herpesviridae*, subfamily *Alphaherpesvirinae*, and genus *Varicellovirus* [1]. It is an important nervous system tropic pathogen in livestock and infects a variety of mammalian species, including ruminants, carnivores, and rodents [2]. Pigs, the natural host of PRV, are a unique animal species that can survive a productive PRV infection and suffer life-long latent infections in the peripheral nervous system [3]. For other susceptible animals, PRV infection is usually fatal [4]. In pigs, the clinical signs of infection vary with the age of the infected individuals. Newborn piglets infected with PRV may develop nervous system disorders and even deaths. For adult pigs however, PRV infection often leads to respiratory diseases. In addition, the virus can even cross the placental barrier of pregnant sows to infect the fetuses and cause abortion [3–5].

Although several countries have eradicated PRV, such as the USA, New Zealand and many members of the European Union, it is still circulating sporadically in many regions all around the globe [6]. Highly pathogenic PRV variants have emerged in numbers of pig farms in China since late 2011 [5, 7–10]. In many large-scale farms, around 50% of pigs were infected, resulting in 3–5% mortality [7, 9]. The driving force behind the high virulence of emerging PRV variants remains unknown. The marketed attenuated live vaccine Bartha-K61 is widely used in the pig industry of China in recent years. Unfortunately, Bartha-K61 cannot confer effective protection against the emerging PRV variants [8, 10]. Thus, the epidemics have the potential to spread outside China to reach the surrounding countries, posing great threats to the pig industry of Asia-Pacific and south-east Asia. Further research on the emerging PRV variant strains is urgently required to control the epidemic situation and further eradicate the disease [11–13].

PRV is a double-stranded DNA virus with a 143-Kb genome containing at least 72 genes [2, 14]. A total of 11 different glycoproteins named gB, gC, gD, gE, gG, gH, gI, gK gL, gM, and gN are distributed in the viral envelope [2]. As the viral fusogen, gB is essential for both viral entry and cell-to-cell spread [15–18]. To induce membrane fusion, the gH-gL heterodimer is required to cooperate with the fusogenic gB [17, 19–21]. Most alphaherpesviruses also require gD to bind receptors and further activate gB to become fusion competent [19, 21, 22].

Similar to other herpesviruses such as herpes simplex virus (HSV) and human cytomegalovirus (HCMV), PRV gB induces protective humoral immunity against viral infection [23–26]. Though PRV has not been reported to infect humans, an antibody raised against PRV gB were observed to cross-react with HSV gB [27], indicating the gBs of different herpesviruses may share some common epitopes. Previously, 26 gB-specific mAbs were identified that cannot directly neutralize PRV *in vitro* but effectively blocked the virus entry in the presence of complement [28]. Further biochemical studies mapped the epitopes of these antibodies to be within three main regions of gB, residues 59–126, 216–279, and 540–734, respectively [29]. Most antibodies target epitopes within residues 540–734 [29]. In order to develop better therapeutics and vaccines, more efforts should be made to characterize the structural and immunogenic properties of gBs of these emerging highly pathogenic PRV variants.

In this work, we immunized mice with soluble PRV gB to generate gB specific mAbs and identified a total of 15 neutralizing antibodies that effectively block PRV entry with either complement dependent or independent mechanisms. To locate their epitopes, we also determined the crystal structure of PRV gB and verified the binding footprints by mutagenesis. These findings would enormously advance our understanding of PRV gB immunogenicity and provide important guidance for antiviral drug design and vaccine development.

## Results

### Identification of PRV neutralizing antibodies targeting the envelope protein gB

We expressed the ectodomain of PRV gB with the Bac-to-Bac expression system. The purified soluble gB protein was then applied to immunize mice. A total of 43 gB-specific mAbs were originally selected by indirect ELISAs from hundreds of hybridoma cells. Virus entry inhibition assay was then conducted to assess the neutralizing activities of these mAbs. The tests were performed with addition of exogenous rabbit complement or without in parallel to identify the potential complement dependent neutralizing activities (See details in Materials and Methods). Fifteen neutralizing mAbs in total were identified, which effectively blocked PRV entry into pig kidney cells (PK-15) (Table 1). Among them, fourteen mAbs blocked the virus entry only in the presence of complement, indicating they exerted the neutralizing activity by complement effect. The remaining one, 1H1 mAb, however directly neutralized the virus without addition of complement (Table 1). Thus, 1H1 mAb might block PRV entry by interfering with either the receptor binding or membrane fusion processes.

**Table 1:** PRV neutralizing activity and protein ELISA reactivities of candidate antibodies.

	Neutralization reactivity		ELISA reactivity	
	Complement^+^	Complement^-^	PRV-gB	PRV-gB-DIV
2-6-G4	**+**	**-**	**+**	**+**
2-5-F4	**+**	**-**	**+**	**+**
2-1-C6	**+**	**-**	**+**	**+**
9A10	**+**	**-**	**+**	**+**
1C10	**+**	**-**	**+**	**+**
1F3	**+**	**-**	**+**	**+**
6D2	**+**	**-**	**+**	**+**
7B11	**+**	**-**	**+**	**+**
9B10	**+**	**-**	**+**	**+**
1-7-C8	**+**	**-**	**+**	**+**
5G12	**+**	**-**	**+**	**+**
6D6	**+**	**-**	**+**	**+**
7E1	**+**	**-**	**+**	**+**
**1H1**	**+**	**+**	**+**	**-**
1H9	**+**	**-**	**+**	**+**

### Broad-spectrum neutralizing activity of 1H1 mAb against different PRV strains

**Fig 1:**
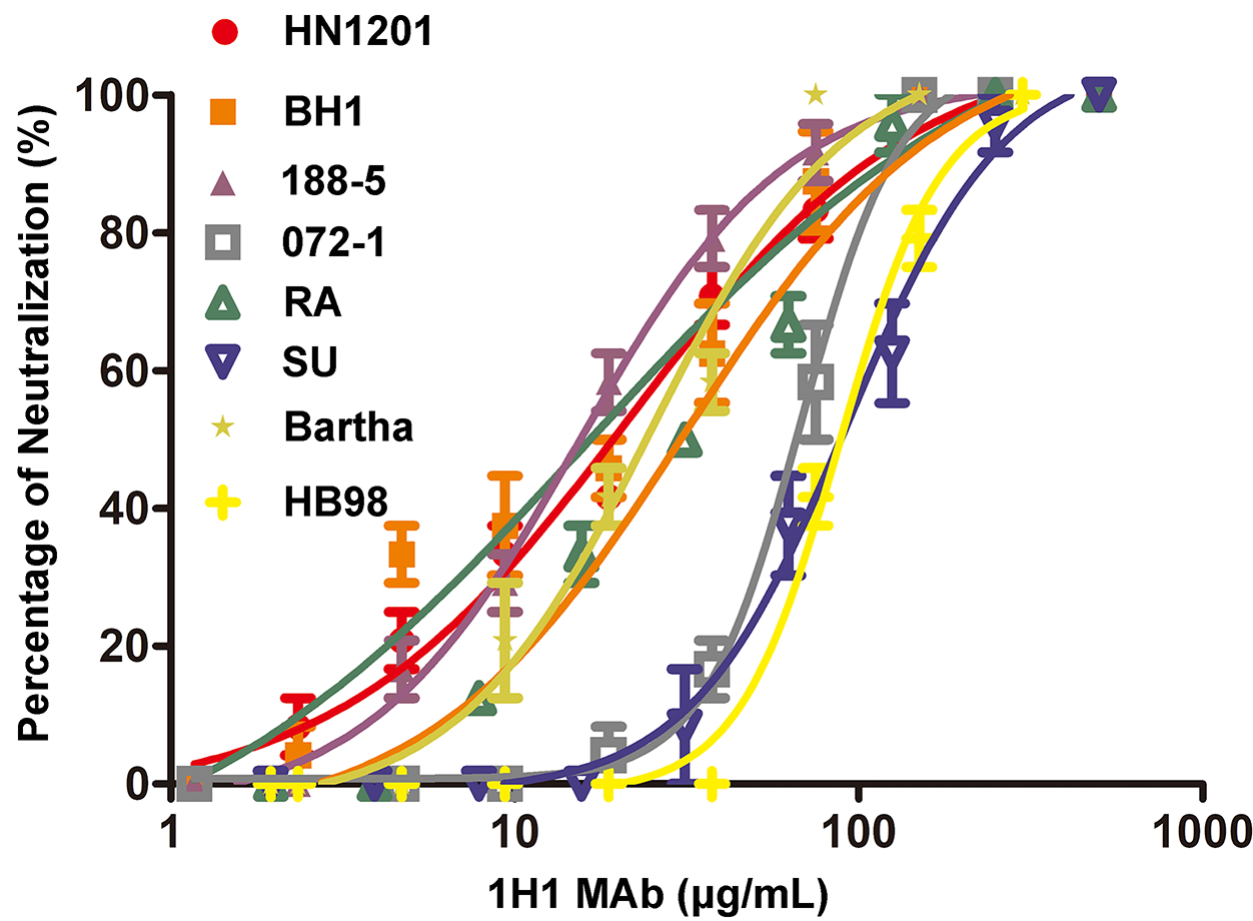
Neutralizing efficacies of 1H1 mAb against different PRV strains. Purified 1H1 mAb was used for virus neutralizing assay to test its efficacy against 8 different PRV strains, including vaccine strains (Bartha and HB98), classical virulent strains (RA and SU), and the current emerging variant strains (HN1201, 188–5, 072–1, and BH1). The neutralizing profile of each strain is represented by different symbols as indicated in the legend. Each experiment was conducted with 3 replicates. The error bars representing standard deviations from the mean are shown correspondingly.

As the only complement-independent neutralizing antibody, we further analyzed the neutralizing efficacies of 1H1 mAb against eight different PRV strains, including vaccine strains (Bartha and HB98), classical virulent strains (RA and SU), and the current emerging variant strains (HN1201, 188–5, 072–1, and BH1). Interestingly, the 1H1 mAb displayed a broad-spectrum neutralizing activity against all PRV strains tested though with varied efficacies (Fig 1). It effectively neutralized HN1201, BH1, 188–5, RA, and Bartha strains, with IC_50_ values ranging from 15.2 to 31.6 μg/mL. In comparison, it is less effective to block the entry of the SU, 072–1, and HB98 strains, for which the IC_50_ values varied from 68.31 to 92.73 μg/mL. As 1H1 mAb targets PRV gB and the gBs of different PRV strains share more than 95% sequence identities (S1 Fig), it is quite conceivable that 1H1 mAb neutralized a broad panel of PRV strains.

### Initial epitope mapping and characterization of these neutralizing antibodies

**Fig 2:**
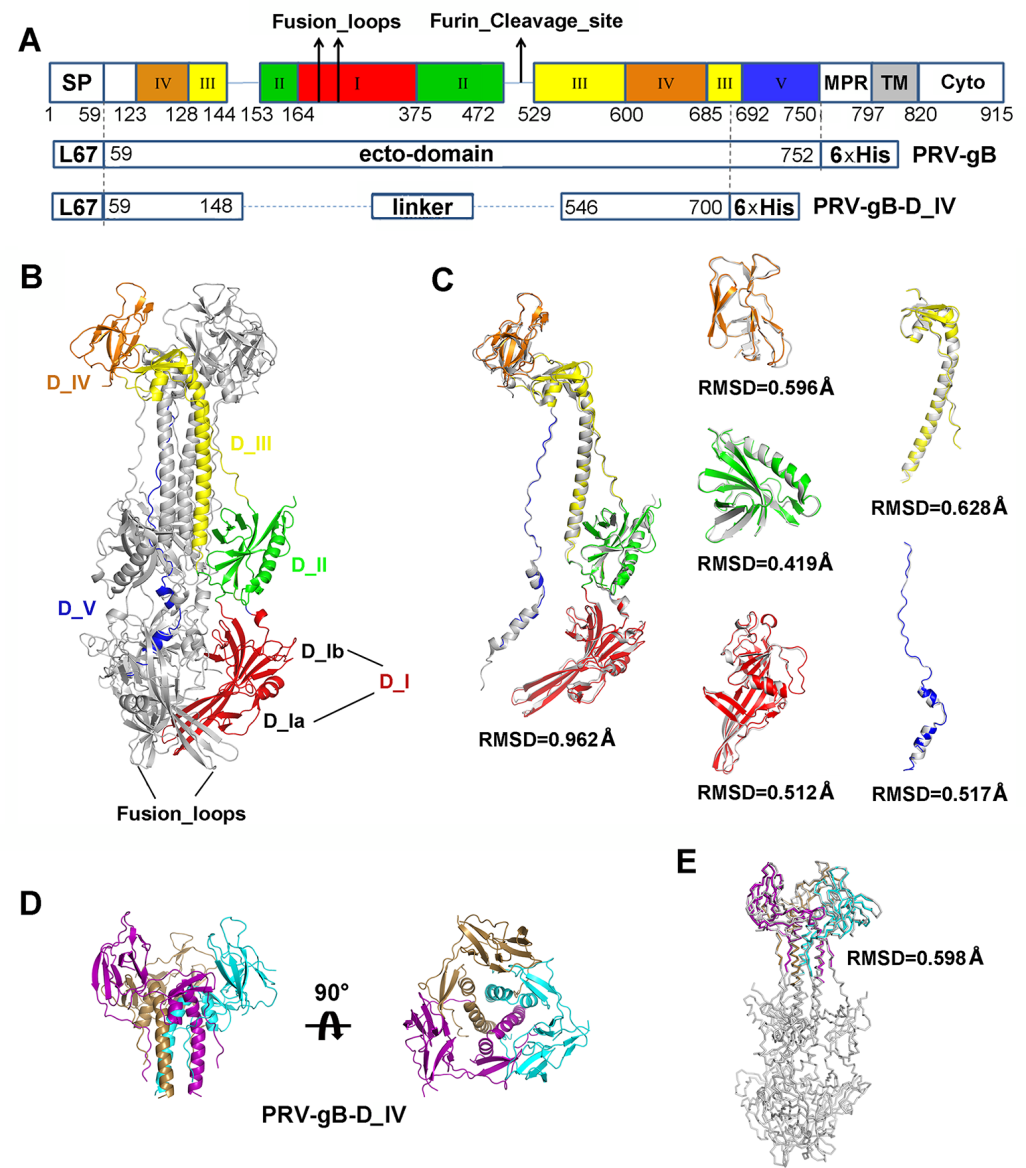
Crystal structure of PRV gB and gB-D_IV. (A) Schematic representation of PRV gB architecture and the construct design of gB ectodomain and D_IV. Each domain is represented by different colors, and the positions of fusion loops and potential furin cleavage site are indicated by arrows. SP, signal peptide; MPR, most polymorphic region; TM, transmembrane region; Cyto, cytoplasmic tail; L67, signal peptide of gp67. (B) Overall structure of PRV gB trimer. One of the protomers is colored by domains as in (A) and labeled accordingly. The fusion loops are indicated by black arrows. (C) Superposition of PRV gB and HSV gB. The gB protomers and each individual domains are superimposed respectively. The rmsd values of Cα atom coordinates of aligned residues are shown under each comparison figure. (D) Crystal structure of PRV gB-D_IV, top and side views. The gB-D_IV exists as homotrimer and is colored by chains. (E) Structural alignment between the PRV gB trimer and D_IV.

Previous studies have indicated residue 540–734 of PRV gB as a hotspot to elicit complement-dependent neutralizing antibodies [29]. As the gBs of HSV and PRV share a sequence identity of ~52% (S2 Fig), we assume that PRV gB would adopt a similar fold as that of HSV gB. In analogy to the structure of HSV gB, residue 540–734 is supposed to be at the upper portion of domain III central helix and the entire domain IV. Thus, we recombinantly expressed a truncated PRV gB protein, denoted as PRV gB-D_IV, which includes the domain IV and a portion of the adjacent domain III central helix (Fig 2A; S3 Fig). With both the soluble PRV gB and PRV gB-D_IV, we tested the binding of all 15 neutralizing antibodies to these two soluble proteins to locate the binding sites. Based on the ELISA experiments, all the 14 complement-dependent neutralizing antibodies can bind to both gB and gB-D_IV (Table 1; S4 Fig), indicating the epitopes of these antibodies are very probably within the domain IV of gB. With these observations and previous reports [29], this region is therefore a suitable immunogen to elicit complement-dependent neutralizing antibodies against PRV infection. In contrast, the 1H1 mAb only reacted with gB but not gB-D_IV (Table 1; S4 Fig), which implied its binding site at other portions of gB beyond the region of gB-D_IV.

To further characterize these neutralizing antibodies, we sequenced the variable regions of all these mAbs. Three pairs of mAbs were found to share the same genes for the variable regions (6D2/9B10, 3E1/7B11, and 1H9/9A10) (Table 2). Thus, we actually obtained 12 unique antibodies, among which 11 of them exert complement-dependent neutralizing activities.

**Table 2:** Sequance characteristics of PRV neutralizing mAbs.

mAb		V-GENE and allele*	J-GENE and allele*	D-GENE and allele*
**1H1**	H chain	IGHV1S81*02	IGHJ2*02	No result
	L chain	IGKV4-74*01	IGKJ1*01	—
**2-5-F4**	H chain	IGHV1-12*01	IGHJ1*01	IGHD1-1*01
	L chain	IGKV4-86*01	IGKJ5*01	—
**3E1**	H chain	IGHV5-9-3*01	IGHJ2*01	IGHD2-3*01
	L chain	IGKV6-23*01	IGKJ5*01	—
**5G12**	H chain	IGHV1S34*01	IGHJ4*01	No result
	L chain	IGKV3-2*01	IGKJ1*01	—
**6D2**	H chain	IGHV1-69*02	IGHJ4*01	IGHD2-14*01
	L chain	IGKV6-23*01	IGKJ1*01	—
**7B11**	H chain	IGHV5-9-3*01	IGHJ2*01	IGHD2-3*01
	L chain	IGKV6-23*01	IGKJ5*01	—
**9B10**	H chain	IGHV1-69*02	IGHJ4*01	IGHD2-14*01
	L chain	IGKV6-23*01	IGKJ1*01	—
**1H9**	H chain	IGHV5-9-1*01	IGHJ2*01	IGHD2-14*01
	L chain	IGKV6-13*01	IGKJ5*01	—
**1-7-C8**	H chain	IGHV5-9-3*01	IGHJ2*01	IGHD4-1*02
	L chain	IGKV6-15*01	IGKJ2*01	—
**1F3**	H chain	IGHV3-2*02	IGHJ2*01	IGHD2-14*01
	L chain	IGKV12-98*01	IGKJ2*01	—
**2-1-C6**	H chain	IGHV5-6-4*01	IGHJ4*01	IGHD1-1*02
	L chain	IGKV6-15*01	IGKJ2*01	—
**2-6-G4**	H chain	IGHV5-12-2*01	IGHJ2*01	IGHD2-10*01
	L chain	IGKV14-111*01	IGKJ1*01	—
**6D6**	H chain	IGHV10-1*02	IGHJ3*01	IGHD4-1*01
	L chain	IGKV14-111*01	IGKJ4*01	—
**7E1**	H chain	IGHV5-17*02	IGHJ1*01	IGHD2-1*01
	L chain	IGKV1-117* 01	IGKJ1*01	—
**9A10**	H chain	IGHV5-9-1*01	IGHJ2*01	IGHD2-14*01
	L chain	IGKV6-13*01	IGKJ5*01	—

* Assignments of sequences were performed with IMGT/V-QUEST (http://www.imgt.org).

### Crystal structures of PRB-gB, PRV-gB-D_IV

In order to further characterize the immunogenic properties of PRV gB and identify the epitopes of these neutralizing antibodies, we determined the crystal structures of PRV gB and gB-D_IV at 3.1 and 2.7 Å resolution, respectively (Fig 2). The overall structures of PRV-gB and HSV gB are highly similar, with a sequence identity of 52% (S2 Fig). The two structures could be well superimposed with an overall rmsd of 0.962 Å (Fig 2C). Similar to other class III viral fusion proteins, PRV gB exists as homotrimers and each protomer could be divided into five domains. Each domain could be superimposed better with their counterpart in HSV gB than the entire gB molecule, as slight domain movement was observed for domain IV relative to the other portion of gB molecule (Fig 2C). In the middle of each gB protomer, a potential furin cleavage site was identified and thus the protein would intend to be processed into two fragments during expression in the cell (Fig 2A). Consistent with this feature, the soluble gB displayed three-band SDS-PAGE profile though eluted as a monodispersed peak in size-exclusion chromatography (S3B Fig). Similar cleavage processing was also observed in HSV gB [15], while it is unclear whether the cleavage is required for the fusogenic activity of gB.

The trimeric gB is mainly stabilized by the central helix bundle formed by domain III in the membrane-distal portion. Domain IV wraps around the top of the central helix bundle to form a crown in the bottle-shaped gB trimer (Fig 2B). The trimerization interfaces are highly stable, such that the truncated gB-D_IV could also assemble into trimers (S3C Fig; Fig 2D). The structure of gB-D_IV trimer could be ideally superimposed with the corresponding portion in the context of the entire gB ectodomain, with a rmsd of 0.598 Å (Fig 2E). The highly stable structure of gB-D_IV further supported the conclusion from our ELISA based assays that these complement-dependent neutralizing antibodies target the crown region of PRV gB and very probably within domain IV.

### The unique glycosylation modification of 1H1 mAb

**Fig 3:**
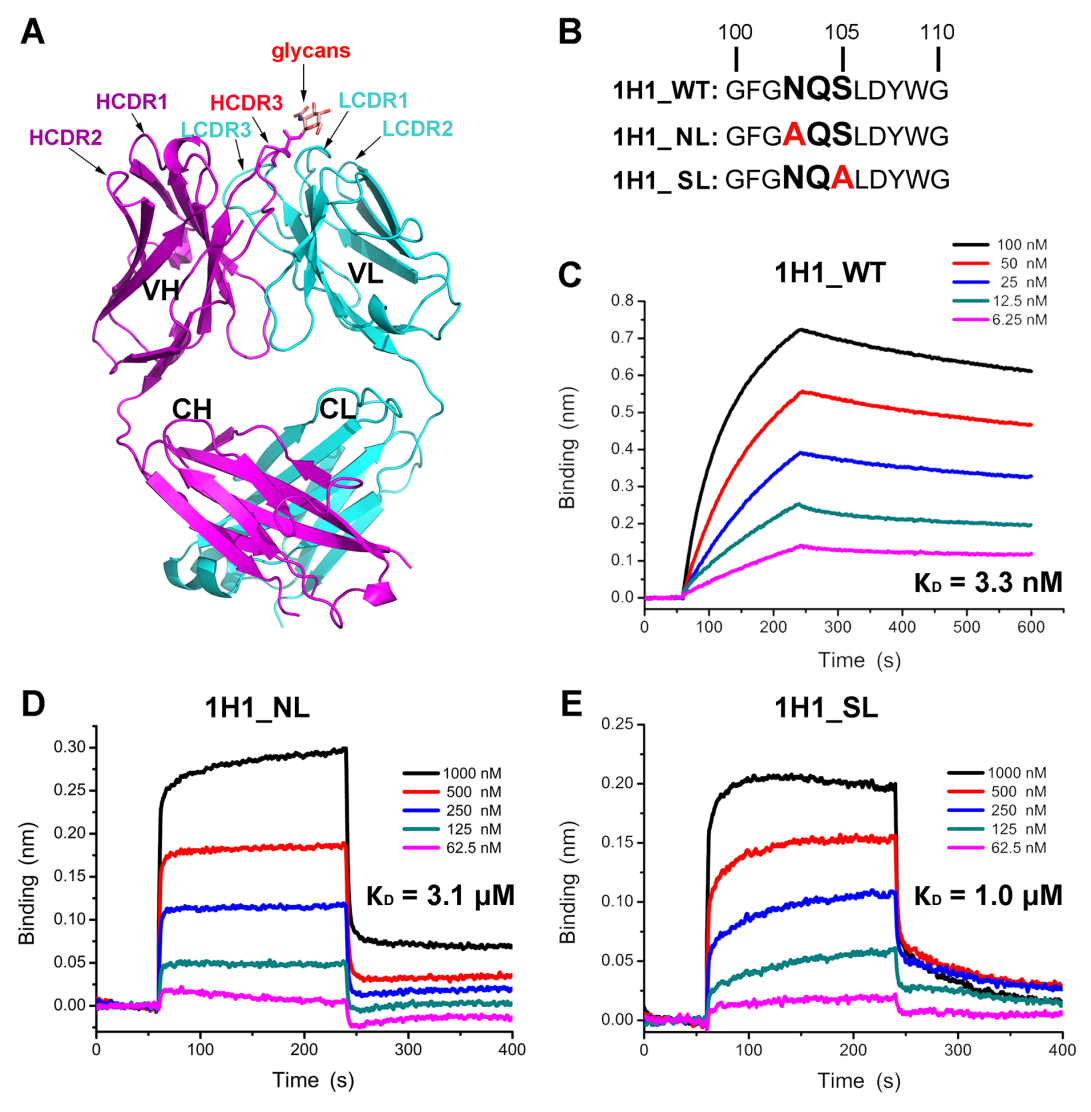
The effect of glycans on antigen recognition of 1H1 Fab. (A) Crystal structure of 1H1 Fab. The heavy chain and light chain are colored in magenta and cyan respectively. The CDR loops are labeled. The HCDR3 and attached glycans are highlighted by red labels. (B) The “NXS” sequence motif, potential N-linked glycosylation sites, in the HCDR3 of 1H1 mAb. The two site mutations to eliminate glycans are highlighted by red. (C-E) The kinetics of wild type and mutant 1H1 Fab binding to PRV gB determined by Octet. The binding curve of each concentration is represented by different colored as the legend. The calculated dissociation constants (K_D_) are shown beside each figure correspondingly.

As the only complement-independent neutralizing antibody identified, the 1H1 mAb recognize PRV gB in regions different from all the other 14 mAbs. To further characterize the antigen recognition properties of this mAb, we solved the crystal structure of 1H1 Fab at a resolution of 2.5 Å (Fig 3A). In this structure, we observed an unusual N-linked glycosylation modification at residue N103 of the HCDR3 loop (S5 Fig). To testify whether the glycans participate in antigen recognition of 1H1 mAb, we introduced site mutations to disrupt the “NXS” motif to eliminate the glycosylation modification (Fig 3B). The mutants were then subjected to bio-layer interferometry (BLI) assay to test their binding affinities to PRV gB. Compared with the wild type 1H1 mAb, the affinities of both the NL and SL mutants decreased almost 1000 times and the binding kinetics also displayed significant differences. The intact 1H1 mAb binds PRV gB with a quite slow kinetics but seems not to dissociate, while the two mutants are much faster in both binding and dissociation processes (Fig 3C–3E). These observations demonstrated that the glycans in the HCDR3 loop play a key but not indispensible role for antigen recognition of 1H1 mAb.

### Identification of the 1H1 binding epitope

**Fig 4:**
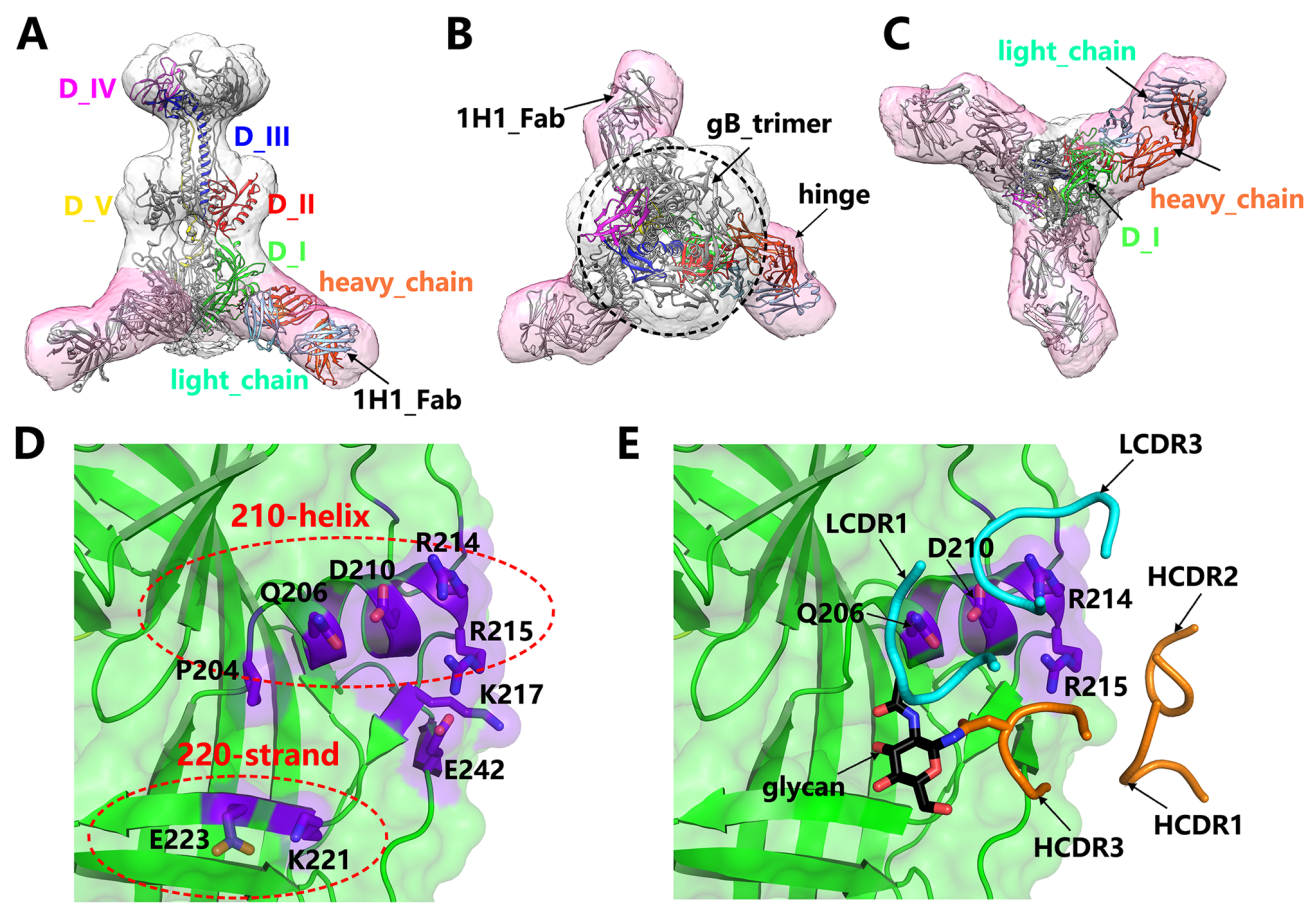
Epitope of 1H1 mAb. (A-C) The EM structure of PRV gB in complex with 1H1 Fab determined by negative stain EM 3D reconstruction. The density map is shown as transparent surfaces and the fitted atomic models are represented by ribbons. The densities corresponding to gB trimer and 1H1 Fab are colored in grey and pink, respectively. The atomic model of one protomer in the PRV gB trimer is colored by domains and labeled accordingly. The heavy chain and light chain of 1H1 Fab are colored in orange and cyan, respectively. This structure clearly shows the binding site of 1H1 Fab in domain I of PRV gB. (D) Potential key residues in the 1H1 epitope on the surface of PRV gB. These residues mainly cluster into two regions, the 210-helix and 220-strand portions. (E) The potential interaction interface between PRV gB and 1H1 Fab. The CDR loops involved in the binding are shown as smooth ribbons and colored by chains as in (A-C). The glycan residue in the HCDR3 is highlighted by a black arrow. The four key residues in the 210-helix region, the main binding footprint, are labeled accordingly.

To precisely identify the epitope of 1H1 mAb, we made great efforts to determine the structure of PRV gB-1H1_Fab complex. Unfortunately, we failed to obtain high quality diffractive crystals of the complex. As an alternative approach, we conducted 3-dimensional (3D) reconstruction by negative stain electron microscopy (EM) method. A 35 Å resolution EM map of PRV gB in complex with 1H1 Fab was obtained (Fig 4; S6 Fig). In this complex, there are three copies of 1H1 Fab binding to the three protomers in the bottle-shaped gB trimer, which follows the rule of 3-fold symmetry (Fig 4A–4C). The atomic structures of PRV gB and 1H1 Fab were perfectly fitted into the density. With the 3-fold symmetry and the hinged structure of 1H1 Fab, we could correctly identify the orientations of heavy chain and light chain (Fig 4A–4C). Thus, a pseudo-atomic model of PRV gB in complex with 1H1 Fab was built so that the interaction details could be inferred.

According to the structure, the epitope of 1H1 was unambiguously mapped to the bottom of domain I in PRV gB (Fig 4A and 4C). The binding of 1H1 Fab to PRV gB is likely mediated by the HCDR3, LCD1 and LCDR3 loops, among which the HCDR3 probably interacts with the 220-strand region and the LCDR1 and LCDR3 mainly recognize the 210-helix (Fig 4D and 4E). We then analyzed all the residues within 5 Å distance in the binding interface to identify the key residues governing the interactions. Among them, four residues in the 210-helix (Q206, D210, R214 and R215) and two in the 220-strand (K221 and E223) seemed to contribute the most interactions, which form a two-portion discrete footprint on the surface of PRV gB (Fig 4D and 4E).

Though only one glycan residue was observed in the density map of 1H1 Fab (Fig 3A), there is probably a long glycan chain attached to N103 in the HCDR3 loop as the glycosylation modification in eukaryotic cells often involves multiple glycan residues. Therefore, the space between the 210-helix and 220-strand could possibly accommodate the glycan chain and the 220-strand is probably involved in the interactions with glycans (Fig 4D and 4E). As glycans play a minor role in the interaction as shown by previous biochemical studies, the main binding footprint would thus fall into the 210-helix region. To further verify the location of 1H1 epitope, we performed mutagenesis on the four candidate residues in the 210-helix to test their effects on the interactions.

**Fig 5:**
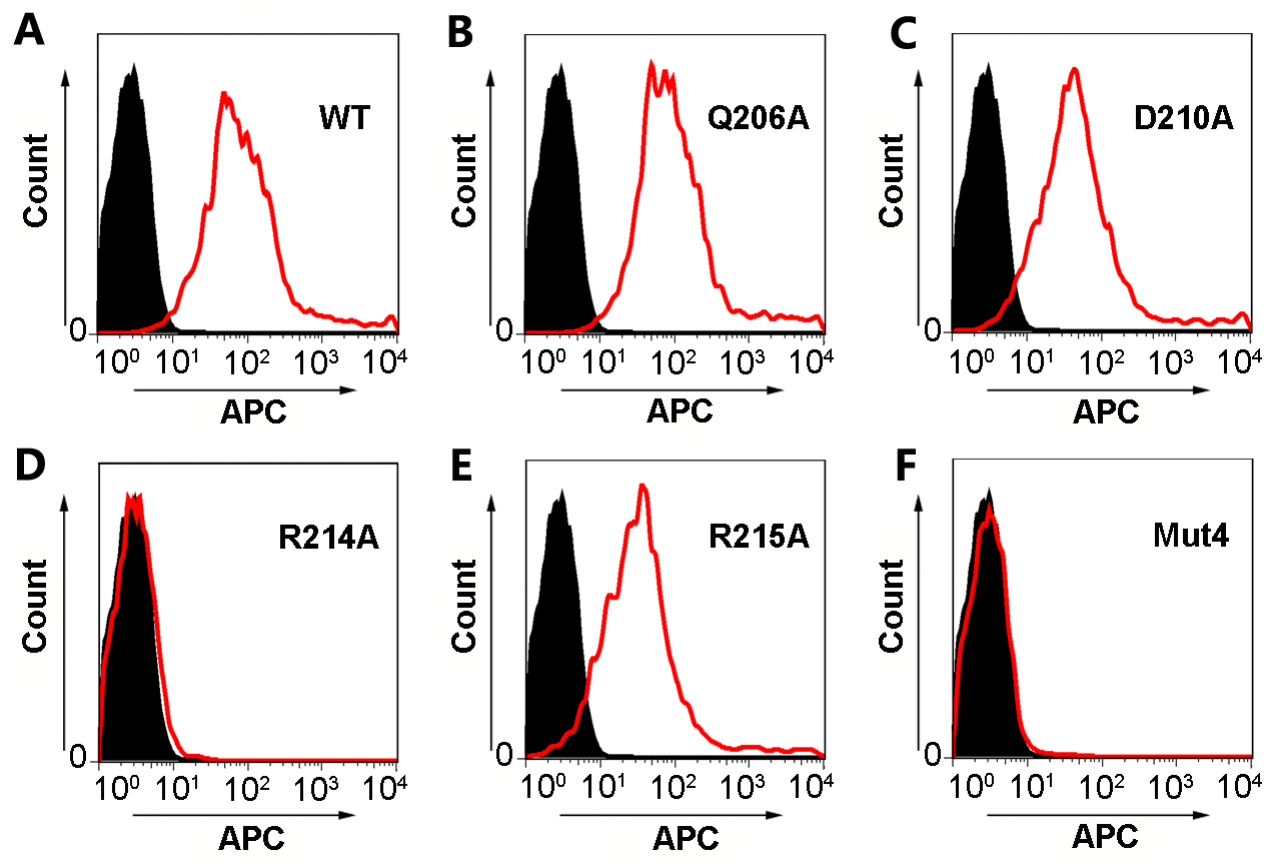
Flow cytometry analysis to verify the 1H1 epitope by mutagenesis. The 293T cells were transfected by wild type (A) PRV gB or mutants (B-F) expression vectors to allow protein expression on the cell surface. The 1H1 mAb was then used to stain the transfected cells, which were further stained by APC-linked secondary antibody. The fluorescence signals of the cells were visualized and quantified by flow cytometry. The profiles of cells transfected with pEGFP-N1 empty vectors are represented by solid black areas (negative control) and the cells transfected with gB or mutant expression plasmids are represented by red silhouettes. WT, wild type; Mut4, quadruple mutants.

The 293T cells were transiently transfected with plasmids encoding full-length wild type or mutant PRV gB and the 1H1 mAb was applied to stain the transfected cells. The binding was visualized and quantified by flow cytometry (Fig 5). As expected, a PRV gB mutant with all the four residues replaced by alanine (Mut4) completely abolished the binding (Fig 5F). Interestingly, Q206A and D210A single mutations displayed no obvious effect on the binding, and mutant R215A only slightly reduced the binding affinity (Fig 5B, 5C and 5E). The R214A single mutation, however, significantly impaired the reactivity of PRV gB to 1H1 Fab (Fig 5D), with the same effect as the quadruple mutant (Mut4). To exclude the possibility that gB mutants failed to be displayed on cell surface due to misfolding, we also stained the cells transfected with gB mutants encoding plasmids by 5G12 mAb which targets the crown region of gB. As shown by the flow cytometry-based assays, all five mutants could be detected on the surface of transfected cells with similar expression levels as the wild type gB (S7 Fig), which demonstrated that the inability of 1H1 mAb to bind gB mutants expressing cells solely results from the substitution of key interacting residues within the epitope. Collectively, these observations implied that the main epitope of 1H1 neutralizing antibody is probably located in the 210-helix region of PRV gB, among which the residue R214 plays a critical role in the interactions.

## Discussion

Outbreaks of newly emerging highly pathogenic PRV variants in Chinese pig farms have caused serious public concerns [5, 8–10]. Poor protective efficacy of marketed vaccines and lack of effective therapeutic drugs further raised the threat that the epidemics might cross the border to affect surrounding countries. Better understanding the structures and antigenic properties of PRV proteins is therefore urgently required for developing effective vaccines and therapeutic drugs.

In common with other members in the *Herpesviridae* family, PRV harbors a pool of glycoproteins embedded in its envelope to form a huge machinery for virus entry, among which gB is the main fusogen responsible for inducing membrane fusion [2, 17, 27]. Previous studies have indicated gB as an effective immunogen to elicit complement-dependent neutralizing antibodies against PRV infections [26, 28, 29]. Our studies further supported the conclusion and identified other antigenic sites to elicit direct neutralizing antibodies as well. Combining our findings with previous reports, we can conclude that domain IV is the immunodominant region of PRV gB. Based on the gB structure we present in this study, domain IV is located on the apexes of the gB trimer to form a "crown", making it fully accessible for both antibody and potential receptor recognition. This domain is quite conceivable to become a hotspot for antibody targeting and thus a suitable candidate to develop subunit vaccines.

It has been established that the entry of herpesviruses involves multiple viral glycoproteins and possibly multiple receptors as well, and gB was also shown to play a role in receptor binding [17]. However, all the antibodies targeting domain IV of gB reported to date do not directly block PRV entry but dependent on the complement effect instead, as shown by cell-based assays *in vitro* [26, 28, 29]. This phenomenon implies that domain IV is probably not a receptor binding site for PRV gB, and that it might retain the same fold before and after membrane fusion with only domain rearrangement in the process of gB conformational changes to mediate membrane fusion. These findings would thus provide important clues to understand the entry mechanisms of PRV and other herpesviruses.

Besides, we also identified a direct neutralizing antibody that effectively blocked the entry of PRV in the absence of complement, 1H1, which targets the domain I of gB. This is the first complement-independent neutralizing epitope of PRV gB reported to date to our knowledge. The 1H1 mAb binds gB at the bottom of domain I, which is very close to the fusion loops. Although the atomic interaction details cannot be elucidated by the low-resolution EM structure, the binding region of 1H1 mAb can be definitely determined, which is also confirmed by the mutagenesis work. Three copies of Fab fragment surround the peripheral of fusion loops, making it probably unable to reach the membrane of host cells. In the context of full-length IgG, the other Fab arm might render extra steric hindrance, which further blocks the interactions between gB and cell membrane. This hypothesis is strongly supported by the observation that gB fusion loops directly interact with lipid bilayer captured by cryo-EM imaging [30]. In analogy to other class III viral fusion proteins, *e*.*g*. the glycoprotein of vesicular stomatitis virus (VSV GP), the domain I of gB might adopt similar fold before and after membrane fusion [31, 32]. Therefore, the 1H1 mAb could probably recognize gB in different conformations, including pre-fusion, post-fusion and the intermediates in between, which is possibly the reason contributing to its high neutralizing efficacy. In addition, these observations also indicate that domain I of gB could possibly serve as an ideal immunogen to elicit direct neutralizing antibodies against herpesviruses.

In summary, we combined both immunological and structural approaches to systematically characterize the envelope protein gB of an emerging highly pathogenic PRV variant. We identified two classes of neutralizing antibodies that effectively block PRV infection *in vitro*, which utilize different mechanisms with complement dependence or without respectively. These two classes of antibodies recognize gB with epitopes in two separate domains and thus indicate these domains as potential subunit vaccines to prevent PRV infections. These findings would intensify our understanding of the immunogenic properties of PRV glycoproteins and provide important guidance for antiviral drug design and vaccine development.

## Materials and methods

### Ethics statement

The protocol in this study was approved by the Committee on the Ethics of Animal Care and Use of National Research Center for Veterinary Medicine (Permit 20160313088). The study was conducted following the Guide for the Care and Use of Animals in Research of the People's Republic of China.

### Cells and viruses

Pig kidney (PK-15) cells (CL33, obtained from ATCC) and African green monkey kidney (Vero) cells (GNO10, obtained from cell resource center of Shanghai Institutes for Biological Sciences, Chinese Academy of Sciences) were cultured in Dulbecco's modified Eagle's medium (DMEM, Gibco) supplemented with 10% fetal bovine serum (Gibco) in a humidified chamber containing 5% CO_2_ at 37°C. PRV strain HN1201 was isolated in 2012 from an affected pig farm in China [5], and was propagated in Vero cells. PRV strains Bartha-K61, HB98, RA, SU, 188–5, 072–1, and BH1 were provided by the National Research Center for Veterinary Medicine, China. To make the current epidemic status and the major molecular changes of PRV clear, a phylogenetic analysis based on all PRV genomes available in the GenBank database was performed using the distance-based neighbor-joining method in MEGA4 software (S8 Fig). The Genbank accession numbers were included in the names of PRV strains.

### Expression and purification of PRV-gB and PRV-gB-D_IV soluble proteins

Both proteins (HN1201 strain [5]) were expressed with the Bac-to-Bac baculovirus expression system (Invitrogen). The PRV gB construct covers nearly the full-length ectodomain, including residues A59 to N752, followed by a C-terminal 6×His tag. The PRV-gB-D_IV includes two continuous regions, residues A59 to S148 and N546 to T700, linked by a GGSG polypeptide, and a 6×His tag is fused at the C terminus. The GP67 signal sequence was engineered at the N-terminus of each protein to facilitate secretion during protein production. To produce recombinant proteins, Hi5 cells were infected with high-titer recombinant baculovirus and grown for 48 h. The cell culture supernatant was harvested by centrifugation applied to metal affinity chromatography with a HisTrap HP column (GE Healthcare). The eluted product was further purified by size-exclusion chromatography using a Superdex 200 16/600 pg column (GE Healthcare) equilibrated with a buffer containing 20 mM Tris–HCl and 150 mM NaCl (pH 8.0). The final product reached a purity of ~95% as shown by SDS-PAGE.

### Generation of gB-specific mAbs

To generate gB-specific mAbs, purified recombinant PRV-gB (100 μg/mouse) was blended with Freund’s complete adjuvant and used to immunize 6-week-old female BALB/c mice. Booster immunizations were performed twice with 3-week intervals. Then the spleen cells were isolated and fused with SP2/0 myeloma cells. Hybridoma culture supernatants were screened for reactivity to purified PRV-gB by standard indirect ELISAs. Positive hybridoma clones were subcloned by limited dilution at least three times. The mAbs were initially captured from hybridoma cell culture supernatants by affinity chromatography with a Protein G HP column (GE Healthcare) and further purified by size-exclusion chromatography.

### Preparation of 1H1 Fab fragment

The coding sequences for 1H1 variable fragments (VH and VL) were fused to the sequence encoding constant regions of a mouse IgG1 (CH, CL and Fc) to generate chimeric IgG1 expression vector using the backbone of pCAGGS plasmid. Two mutant protein-expressing plasmids (1H1_NL and 1H1_SL) were constructed by site-directed mutagenesis to replace N103 or S105 in HCDR3 with alanine (Fig 3B). The plasmids were transiently transfected into human embryonic kidney 293T (HEK293T) cells for protein expression. After three to seven days post transfection, the supernatants containing secreted IgG1s were harvested and subjected to HiTrap ProteinG chromatography (GE Healthcare). Target proteins were eluted with 0.1 M glycine (pH 3.0) and further purified by size-exclusion chromatography using a Superdex 200 16/600 pg column (GE Healthcare). Fab fragments were generated by ficin digestion and purified using the pierce mouse IgG1 Fab preparation kit (Thermo Scientific) following the manufacturer’s instructions. The products were buffer-exchanged into a buffer containing 20 mM Tris-HCl (pH 8.0), 50 mM NaCl by an additional round of size-exclusion chromatography for crystallization.

### Indirect ELISAs

Briefly, 96-well microtiter plates were coated with purified PRV-gB and PRV-gB-D_IV at 200 ng/well in a carbonate-bicarbonate coating buffer (pH 9.6) at 4°C overnight. Plates were blocked at 37°C for 1 h with PBST containing 5% skimmed milk. Antibodies were then added in the well and incubated for 1 h at 37°C. After three times of washing, the wells were incubated with goat anti-mouse IgG-HRP (Santa Cruz) for 40 min at 37°C. The plates were washed again for five times before the reaction substrate TMB was added. The reaction was conducted in dark at room temperature for 5 min and was stopped with 2 M H_2_SO_4_. The optical density at 450 nm (OD_450_) of each well was read using a microplate reader (Thermo). Statistical presentations were generated with GraphPad Prism 5 (San Diego, CA).

### Virus neutralization assays in the presence or absence of complement

PK-15 cells were seeded in 96-well plates. Pseudorabies viruses (200 times of TCID_50_) were incubated with serial two-fold dilutions of the 1H1 mAb at 37°C for 1 h prior infecting cells. Then, the mixture was added to PK-15 monolayers in 96-well plates and incubated for 1 h. Each concentration was conducted with eight replicates. The supernatant was removed after incubation and replaced by fresh DMEM medium. The cells were cultivated for another 72 h at 37°C before analysis. The cellular pathology was directly observed using microscopy. All experiments were conducted in three independent trials. The half maximal inhibitory concentration (IC_50_) was measured to describe the neutralization titer of each antibody. To identify complement-dependent neutralization activities, the experiment was conducted following the same protocol as above except that fresh rabbit serum (working concentration: 5%) as an exogenous complement was added into the virus-antibody mixture before infecting the cells. The data was graphed using GraphPad Prism 5 for presentation (San Diego, CA).

### Sequencing of the immunoglobulin variable regions of hybridoma cells

The V gene sequences of each mAb clone were amplified as previously described [33]. Briefly, 10^6^ hybridoma cells were collected by centrifugation. Total RNA was extracted using TRIzol regent (Takara) according to the manufacturer’s protocol. Reverse transcription and PCR amplification were performed using a set of primers [33]. PCR products were identified by agarose gel electrophoresis and purified using a commercial kit (Tiangen). The DNA fragments were cloned into the pMD 18-T vector (Takara) and sequenced individually.

### Crystallization, data collection, and structure determination

The protein samples were concentrated to 10 mg/mL for crystallization using the sitting drop vapor diffusion method at 18°C. PRV gB was crystallized with a reservoir solution of 34% PEG200 and 0.1 M citric acid, pH 6.5. The crystals of PRV-gB-D_IV were obtained in reservoir solution containing 0.05 M calcium chloride dihydrate and 0.1 M MES, pH 6.0. The 1H1 Fab was crystallized with a reservoir solution of 0.2 M potassium sulfate and 20% PEG3350, pH 6.8. X-ray diffraction data was collected at the Shanghai Synchrotron Radiation Facility (SSRF) BL17U at a wavelength of 0.97915 Å [34]. The datasets were processed with HKL2000 software [35]. Structures were determined by the molecular replacement method using the Phaser program [36] in the CCP4 suite [37]. The PRV gB structure was solved using the HSV-1 gB structure (PDB ID: 2GUM) as the search model. The Fab structure (PDB ID: 1SY6) was used as the search input for 1H1 Fab structure determination. Initial restrained rigid-body refinement was performed using PHENIX [38], which was followed by manual rebuilding and adjustment in COOT [39]. Further refinement was performed using PHENIX [38]. The stereochemical qualities of the final models were assessed using MOLPROBITY [40]. All the data collection and refinement statistics are summarized in S1 Table.

### Bio-layer interferometry (BLI)

The binding affinities of wild type 1H1 Fab or mutants to PRV gB were measured by BLI at room temperature (298K) with the Octet RED96 biosensor method (ForteBio, Inc.). The runnning buffer is composed of 20 mM Hepes (pH 7.4), 150 mM NaCl and 0.005% (vol/vol) Tween 20. Soluble gB was immobilized on an Ni-NTA-coated biosensor surface and then exposed to a series of analytes at different concentrations (6.25–100 nM for 1H1_WT, 62.5–1000 nM for 1H1_NL or 1H1_SL). Background subtraction was used to correct the errors of sensor drifting. The data was processed by the ForteBio’s data analysis software and plotted with Origin 8.0 program.

### Negative stain electron microscopy and 3D reconstruction

To prepare the PRV gB-1H1 Fab complex, soluble PRV gB and 1H1 Fab samples were mixed with a molar ratio of 1:1.5 and incubated at 4°C for 2 h. The mixture was then separated by size-exclusion chromatography using a Superose 6 10/300 GL column (GE Healthcare). The complex sample at a concentration of 0.02 mg/mL was applied to glow-discharged copper grids coated with continuous carbon films and stained with 2% uranyl acetate. The excessive stain liquor was blotted with a filter paper and let the grid to air-dry. The specimen was then loaded onto a Tecnai F20 transmission electron microscope (FEI) equipped with a field emission gun for data collection, which was operated at 200 kV acceleration voltage and with a defocus range of—(1–3) μm. Images were recorded with a 4k×4k BM-Eagle CCD camera with a calibrated pixel size of 1.36 Å.

A total of ~8000 particles were semi-automatically picked from 200 micrographs using e2boxer.py in EMAN2 [41] package. The contrast transfer function (CTF) parameters were estimated by e2ctffit.py [41] and applied to correct the images by phase-flipping [42] method. All the subsequent classification and reconstruction processes were conducted with relion-2.0 [43] using the phase-flipped particles without further CTF corrections. After several rounds of iterative 2D and 3D classifications, a stack of ~3000 particles was selected with 3 copies of 1H1 Fab bound to a gB trimer. The stack was subjected to 3D refinement with 3-fold symmetry applied, which resulted in a reconstruction of 35 Å resolution as determined by the gold-standard fourier shell correlation (FSC) 0.5 cut-off value (S6 Fig).

### Map interpretation and atomic model fitting

Though with low resolution, the reconstructed map clearly shows the feature of gB trimer in the center and three copies of 1H1 Fab density branching out at one end. We first fitted the crystal structure of PRV gB into the density map using CHIMERA [44], which showed a high degree of matching. The atomic structure of 1H1 Fab was further fitted into the remaining density using SITUS [45] with several rounds of orientation search. The special hinged structure of the Fab density allowed us to distinguish the relative orientation of heavy chain and light chain of Fab molecules despite the low resolution of the reconstruction. All the fitting processes were performed following the rigid-docking protocols without local adjustments. No obvious clash or close contact was observed in the final pseudo-atomic model, which was used for further structural analysis. All the EM density related figures were rendered using CHIMERA [44].

### Mutagenesis and flow cytometric analysis

The gene sequence encoding the full length wild type PRV gB (amino acids 1–914) was cloned into the vector pEGFP-N1 to generate a gB expression vector with an EGFP tag fused at the C-terminus. A QuickChange site-directed mutagensis kit was used to obtain the mutants with the indicated mutations (Q206A, D210A, R214A, R215A or Mut4). Mut4 denotes the quadruple mutant with all four amino acids replaced by Alanine. Protein expression was verified by fluorescence microscopy.

The binding between the 1H1 mAb and gB/mutants was analyzed by flow cytometry. Briefly, Human Embryonic Kidney 293 cells with large T antigen (293T cells, obtained from cell resource center of Shanghai Institutes for Biological Sciences, Chinese Academy of Sciences) were transfected with these plasmids above separately. After 24 h, the transfected cells were incubated with 1H1 mAb at room temperature for 30 min. The cells were then washed 3 times with 1×PBS to remove the unbound antibodies. Subsequently, the cells were further incubated with APC-linked goat anti-mouse IgG (minimal x-reactivity) (Biolegend, U.S.A) secondary antibody for 30 min at room temperature (avoiding light). Again, discard the liquid and wash 3 times with 1×PBS. Finally, the cells were loaded onto the flow cytometry (BD FACSCalibur) to detect the APC fluorescence signals.

## Supporting information

S1 Table Data collection and refinement statistics. (DOCX)

S1 Fig Amino acid sequence alignment of gBs from different PRV strains. The gB sequences of all the PRV strains analyzed in Fig 1 are aligned, which shows more than 95% sequence identity. The potential key residues in the 1H1 epitope are highly conserved among all PRV strains as indicated by blue pentagrams.
(TIF)

S2 Fig Amino acid sequence alignment of HSV-1 gB with PRV gB. The sequence of HSV-1 gB (GenBank accession number: ABM52972.1) and PRV gB (GenBank accession number: ALT14233.1) are aligned. The positions of fusion loops and furin cleavage site are indicated by arrows and labeled aside correspondingly. The potential key residues in the 1H1 epitope are highlighted by blue pentagrams.
(TIF)

S3 Fig Size-exclusion chromatograms and SDS-PAGE profiles of PRV gB and gB-D_IV soluble proteins. (A) A standard elution profile for molecular weight calibration. The five standard samples used in this analysis are given at the right side with the molecular weights labeled accordingly. Both gB (B) and gB-D_IV (C) exist as trimers in solution estimated by the elution volumes. The SDS-PAGE profile of gB shows three bands, corresponding to the full-length gB (gBa) and furin cleaved products (gBb and gBc), respectively. The gB-D_IV shows a single band in the SDS-PAGE profile, indicating high stability of this truncated protein.
(TIF)

S4 Fig Reactivities of the 15 neutralizing antibodies to PRV gB and gB-D_IV determined by ELISA. The abscissa and ordinate represent the OD_450_ values of antibodies reacting to plates coated with gB-D_IV and gB ectodomain, respectively. Each antibody is represented by a blue spot and labeled aside correspondingly.
(TIF)

S5 Fig The electron density of glycan residue in the HCDR3 loop of 1H1 Fab. The 1H1 Fab is shown as cartoon and colored by chains (heavy chain: magenta; light chain: cyan). The side chain of N103 (HCDR3) and attached glycan residue are shown as sticks and colored by elements. The electron density (2Fo-Fc map, at 1.0 σ contour level) of the glycan residue is shown as black meshes.
(TIF)

S6 Fig Negative stain EM analysis and 3D reconstruction of PRV gB in complex with 1H1 Fab. (A) A representative negative stain micrograph of gB-1H1_Fab complex. (B) Typical 2D class average images of the complex, top and side views. The density corresponding to Fab molecules are indicated by red arrows. (C) Fourier shell correlation (FSC) curve of the final reconstruction. The gold-standard 0.5 cut-off value is indicated by blue dashed lines, which corresponds to a resolution of 35 Å. (D) Euler angle distribution of the final reconstruction shown at both top and side views. The 3-fold axis of the complex is indicated by a black triangle.
(TIF)

S7 Fig Flow cytometry analysis to detect the surface display of PRV gB mutants in transfected cells. The 293T cells were transfected with either WT gB or mutant expression vectors. The transfected cells were first stained by 5G12 mAb and then the APC-linked secondary antibody was applied for detection by flow cytometry. Cells transfected with pEGFP-N1 empty vectors (negative control) are represented by solid black areas, and those transfected with WT gB (A) or mutants (B-F) expression plasmids are shown as red silhouettes in each panel.
(TIF)

S8 Fig Phylogenetic analysis of all PRV genomes available in the GenBank database. The analysis was performed by using the distance-based neighbor-joining method in MEGA4 software. The Genbank accession numbers are included in the names of all taxons.
(TIF)
