# Supplementary material for: Two classes of protective antibodies against Pseudorabies virus variant glycoprotein B: Implications for vaccine design
Source: PLoS Pathog. 2017 Dec 20;13(12):e1006777. doi: 10.1371/journal.ppat.1006777 (PMC5754140; doi:10.1371/journal.ppat.1006777)
Supplement: S1 Table — (DOCX) [file ppat.1006777.s001.docx]

**Table S1. Data collection and refinement statistics**

|  | **PRV-gB** | **PRV-gB-DIV** | **1H1 Fab** |
| --- | --- | --- | --- |
| **Data statistics** |  |  |  |
| Space group | H3 | P22_1_2_1_ | P1 |
| Cell dimensions |  |  |  |
| a, b, c (Å) | 100.29, 100.29, 272.92 | 91.911, 119.850, 123.771 | 64.18, 81.09, 98.47 |
| α, β, γ (°) | 90, 90, 120 | 90, 90, 90 | 85.68, 82.85, 71.45 |
| Resolution (Å) ^a^ | 50-3.1 (3.2-3.1) | 50-2.7 (2.8-2.7) | 42-2.5 (2.6 - 2.5) |
| Completeness (%) ^a^ | 99.8 (99.9) | 99.6 (99.5) | 98.3 (98.0) |
| Redundancy ^a^ | 5.1 (5.1) | 4.4 (4.5) | 2.9 (3.0) |
| R_merge_ ^b^ | 0.103 (0.725) | 0.094 (0.847) | 0.083 (0.300) |
| I/σI ^a^ | 15.779 (2.716) | 15.078 (1.803) | 13.326 (3.026) |
| **Refinement** |  |  |  |
| Resolution (Å) | 35.57-3.09 | 46.52 - 2.70 | 41.63 - 2.48 |
| No.Reflections (free) | 18746 (1856) | 38147 (1913) | 63867 (5811) |
| Rwork/Rfree ^c^ | 0.2208/0.2628 | 0.2267/0.2911 | 0.2032/0.2564 |
| No. atoms |  |  |  |
| Protein | 4582 | 8134 | 12942 |
| Ligands | 0 | 0 | 56 |
| B-factors |  |  |  |
| Protein | 96.63 | 46.54 | 48.58 |
| Glycans | - | - | 78.47 |
| R.m.s.deviations. |  |  |  |
| Bond lengths (Å) | 0.012 | 0.010 | 0.010 |
| Bond angles (º) | 1.35 | 1.39 | 1.18 |
| Ramachandran plot ^d^ |  |  |  |
| Favored (%) | 88.7 | 92.9 | 95.0 |
| Allowed (%) | 8.8 | 7.1 | 4.3 |
| Outliers (%) | 2.5 | 0.8 | 0.8 |

^a^ Values for the outmost resolution shell are given in parentheses.

^b^ R_merge_=Σ_i_Σ_hkl_| I_i_<I> | /Σ_i_Σ_hkl_I_i_, where I_i_ is the observed intensity and <I> is the average intensity from multiple measurements.

^c^ R_work_ = Σ | | F_o_| | F_c_ | | /Σ | F_o_| , where F_o_ and F_c_ are the structure-factor amplitudes from the data and the model, respectively. R_free_ is the R factor for a subset of reflections that was selected prior to refinement calculations and was not included in the refinement.

^d^ Ramachandran plots were generated by using the program MolProbity.
