# Supplementary figures and images for: Two classes of protective antibodies against Pseudorabies virus variant glycoprotein B: Implications for vaccine design
Source: PLoS Pathog. 2017 Dec 20;13(12):e1006777. doi: 10.1371/journal.ppat.1006777 (PMC5754140; doi:10.1371/journal.ppat.1006777)

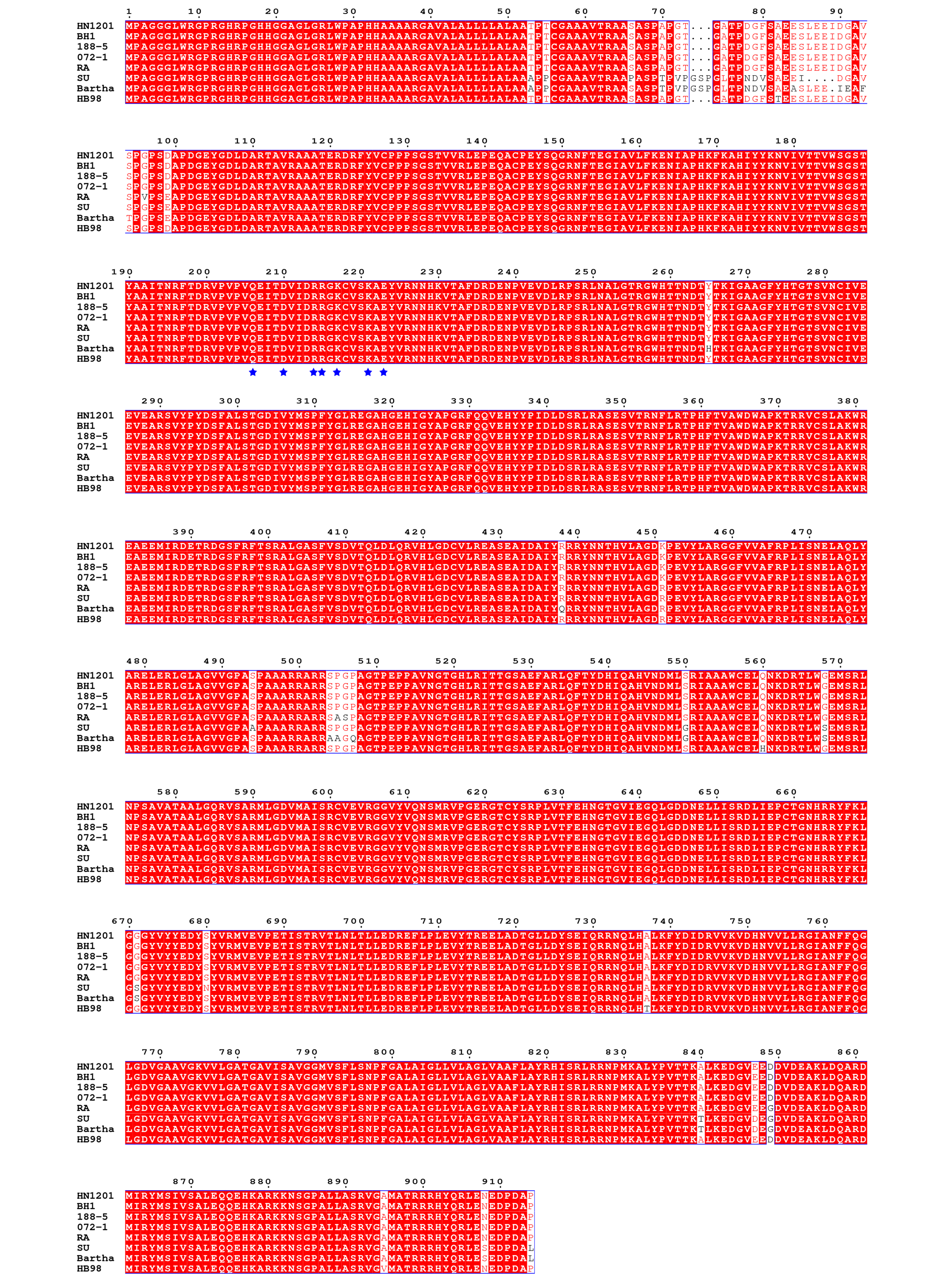

Supplement: S1 Fig — The gB sequences of all the PRV strains analyzed in Fig 1 are aligned, which shows more than 95% sequence identity. The potential key residues in the 1H1 epitope are highly conserved among all PRV strains as indicated by blue pentagrams. (TIF) [file ppat.1006777.s002.tif]

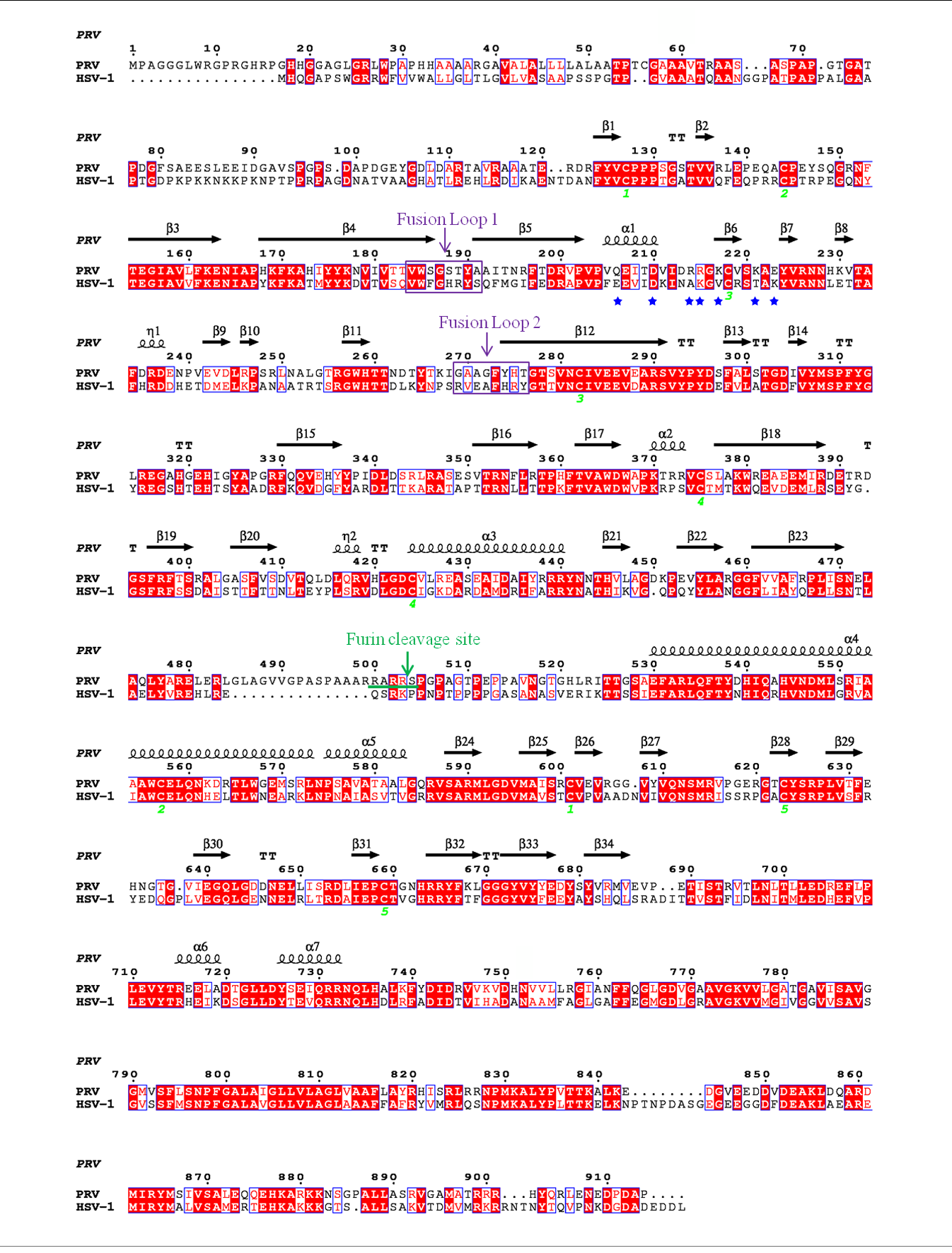

Supplement: S2 Fig — The sequence of HSV-1 gB (GenBank accession number: ABM52972.1) and PRV gB (GenBank accession number: ALT14233.1) are aligned. The positions of fusion loops and furin cleavage site are indicated by arrows and labeled aside correspondingly. The potential key residues in the 1H1 epitope are highlighted by blue pentagrams. (TIF) [file ppat.1006777.s003.tif]

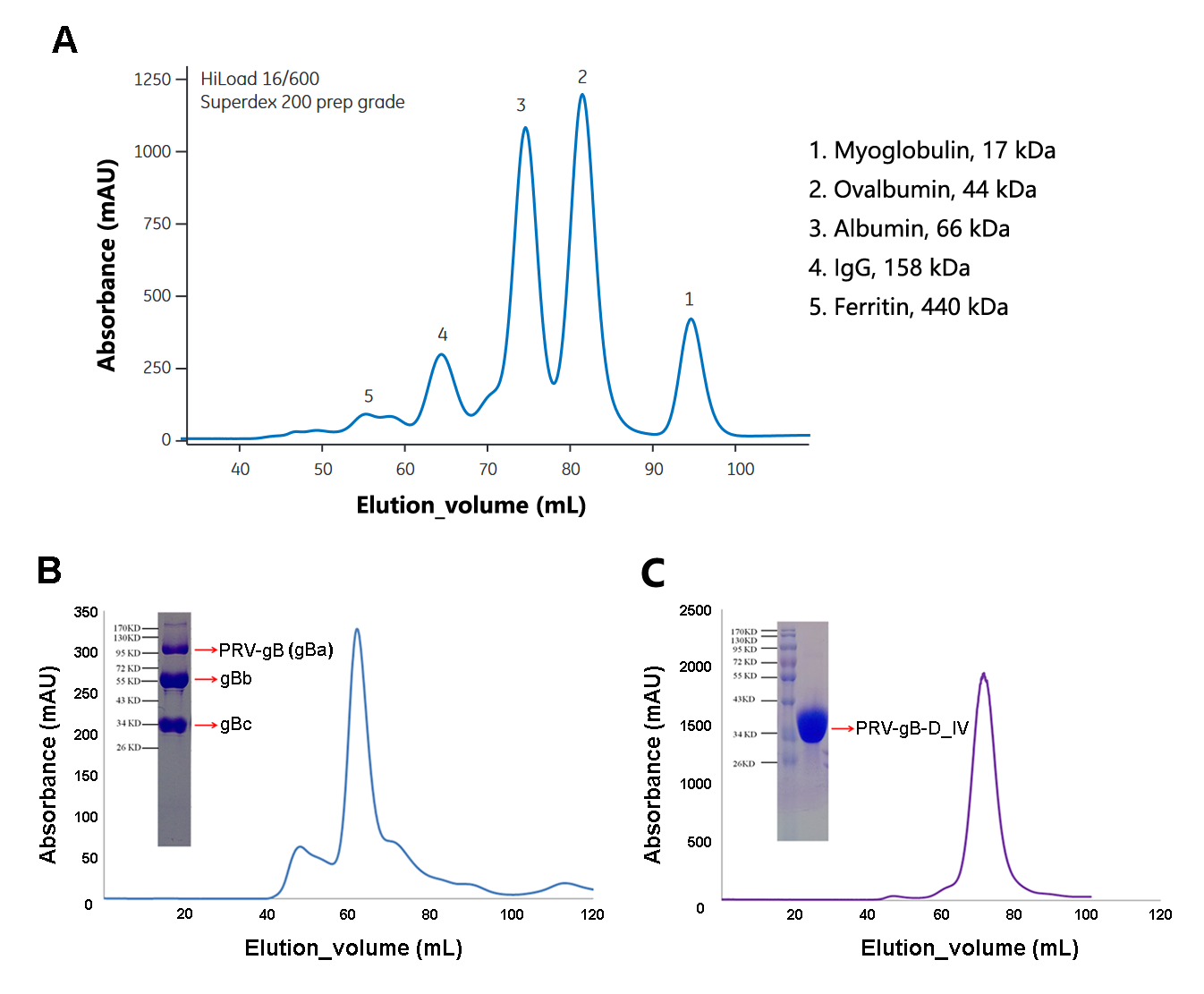

Supplement: S3 Fig — (A) A standard elution profile for molecular weight calibration. The five standard samples used in this analysis are given at the right side with the molecular weights labeled accordingly. Both gB (B) and gB-D_IV (C) exist as trimers in solution estimated by the elution volumes. The SDS-PAGE profile of gB shows three bands, corresponding to the full-length gB (gBa) and furin cleaved products (gBb and gBc), respectively. The gB-D_IV shows a single band in the SDS-PAGE profile, indicating high stability of this truncated protein. (TIF) [file ppat.1006777.s004.tif]

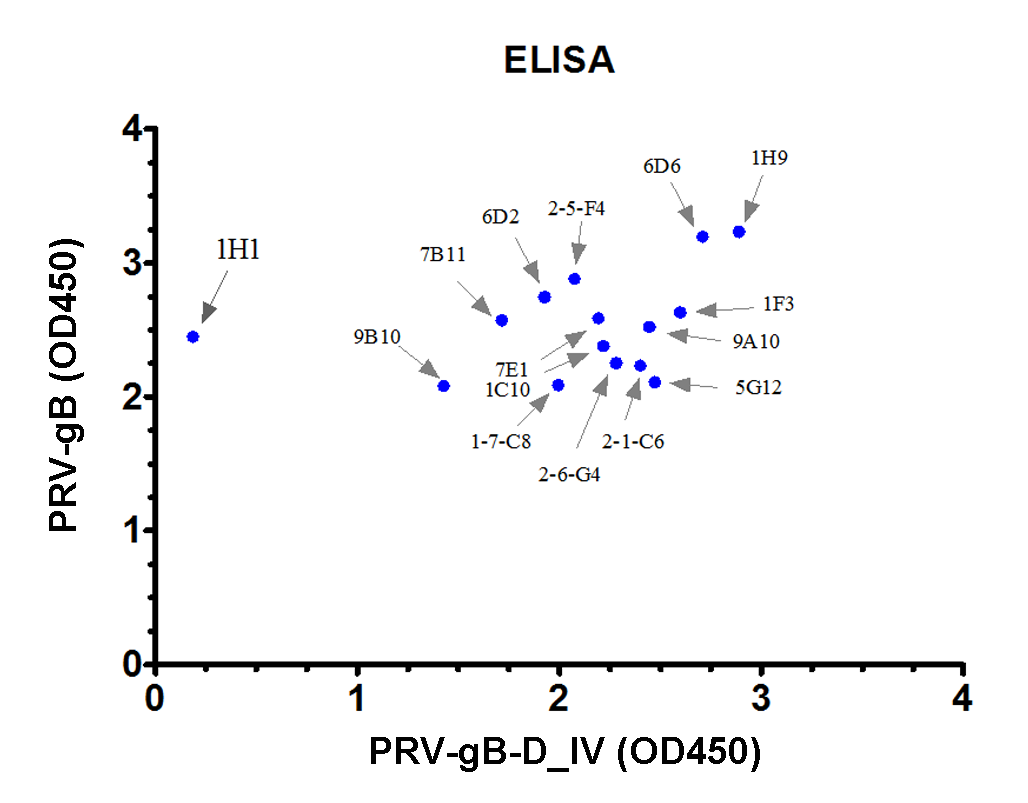

Supplement: S4 Fig — The abscissa and ordinate represent the OD450 values of antibodies reacting to plates coated with gB-D_IV and gB ectodomain, respectively. Each antibody is represented by a blue spot and labeled aside correspondingly. (TIF) [file ppat.1006777.s005.tif]

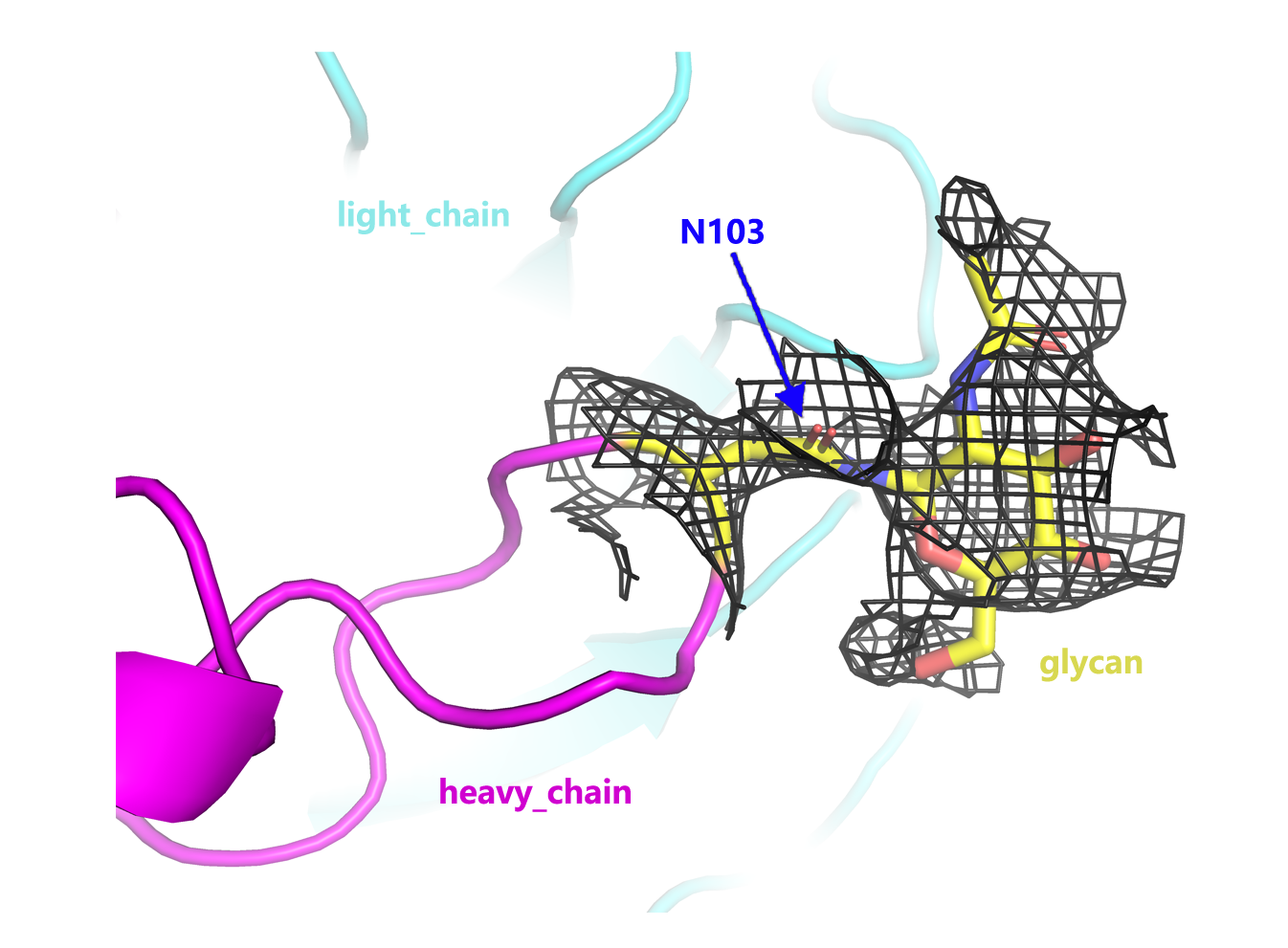

Supplement: S5 Fig — The 1H1 Fab is shown as cartoon and colored by chains (heavy chain: magenta; light chain: cyan). The side chain of N103 (HCDR3) and attached glycan residue are shown as sticks and colored by elements. The electron density (2Fo-Fc map, at 1.0 σ contour level) of the glycan residue is shown as black meshes. (TIF) [file ppat.1006777.s006.tif]

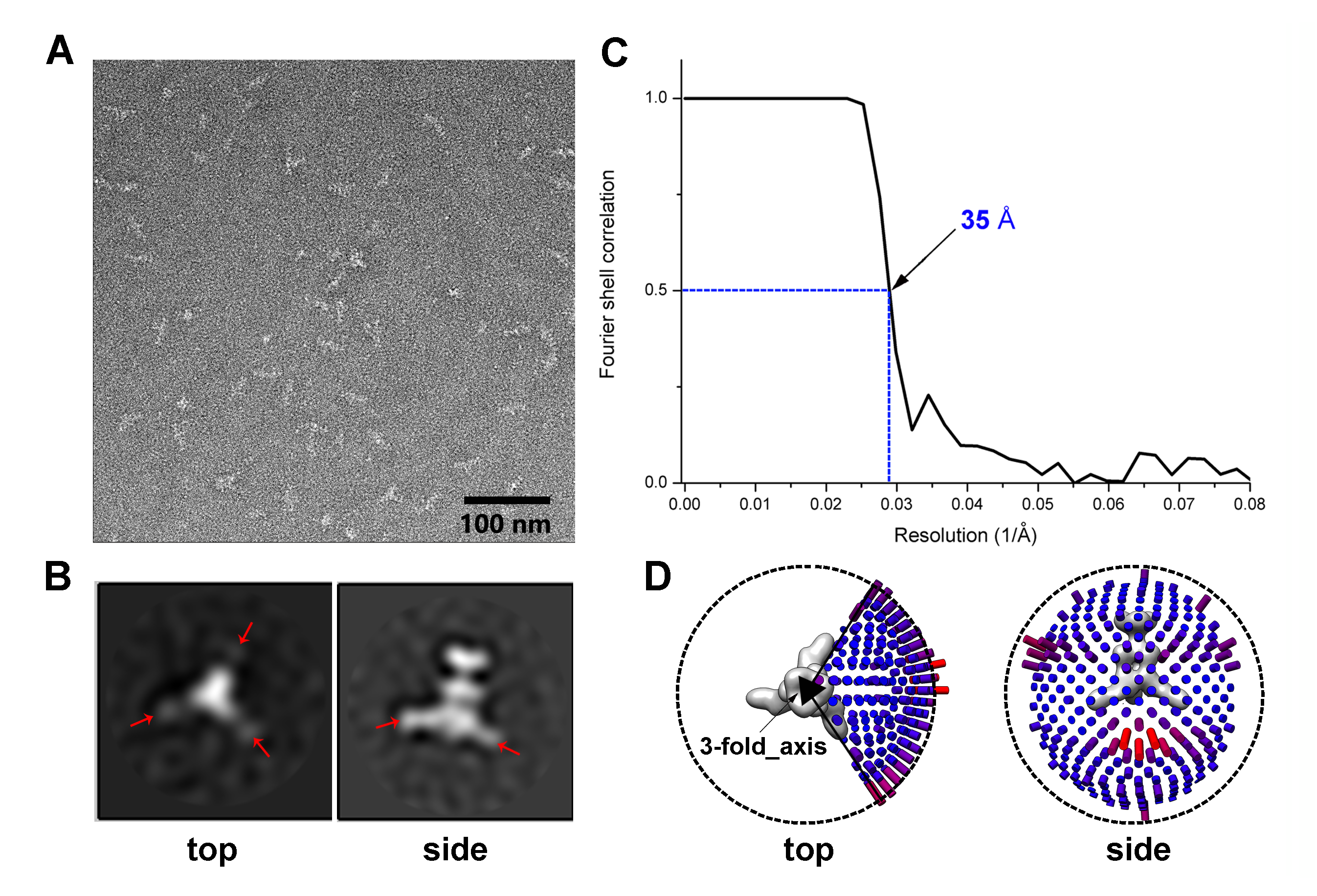

Supplement: S6 Fig — (A) A representative negative stain micrograph of gB-1H1_Fab complex. (B) Typical 2D class average images of the complex, top and side views. The density corresponding to Fab molecules are indicated by red arrows. (C) Fourier shell correlation (FSC) curve of the final reconstruction. The gold-standard 0.5 cut-off value is indicated by blue dashed lines, which corresponds to a resolution of 35 Å. (D) Euler angle distribution of the final reconstruction shown at both top and side views. The 3-fold axis of the complex is indicated by a black triangle. (TIF) [file ppat.1006777.s007.tif]

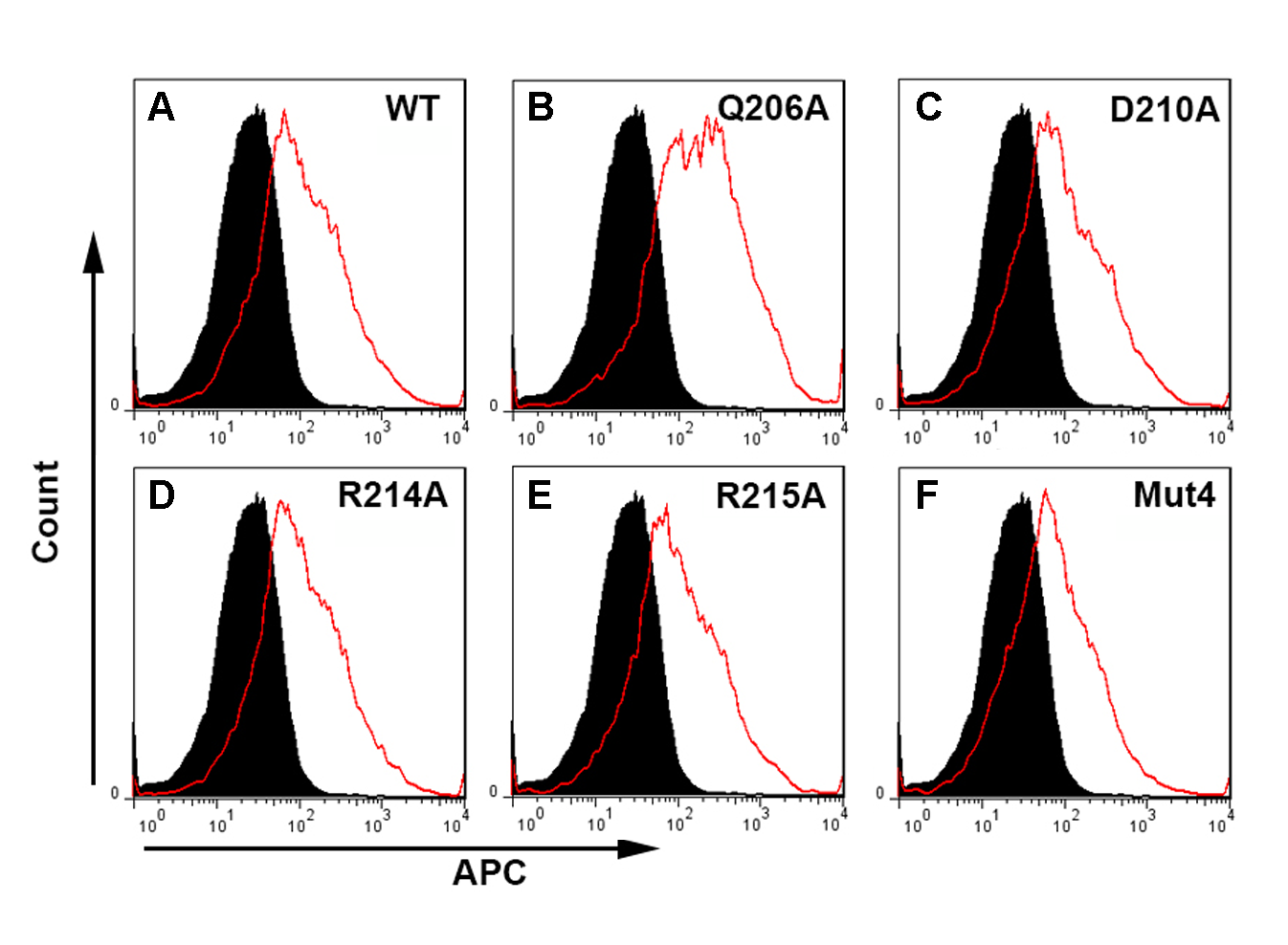

Supplement: S7 Fig — The 293T cells were transfected with either WT gB or mutant expression vectors. The transfected cells were first stained by 5G12 mAb and then the APC-linked secondary antibody was applied for detection by flow cytometry. Cells transfected with pEGFP-N1 empty vectors (negative control) are represented by solid black areas, and those transfected with WT gB (A) or mutants (B-F) expression plasmids are shown as red silhouettes in each panel. (TIF) [file ppat.1006777.s008.tif]

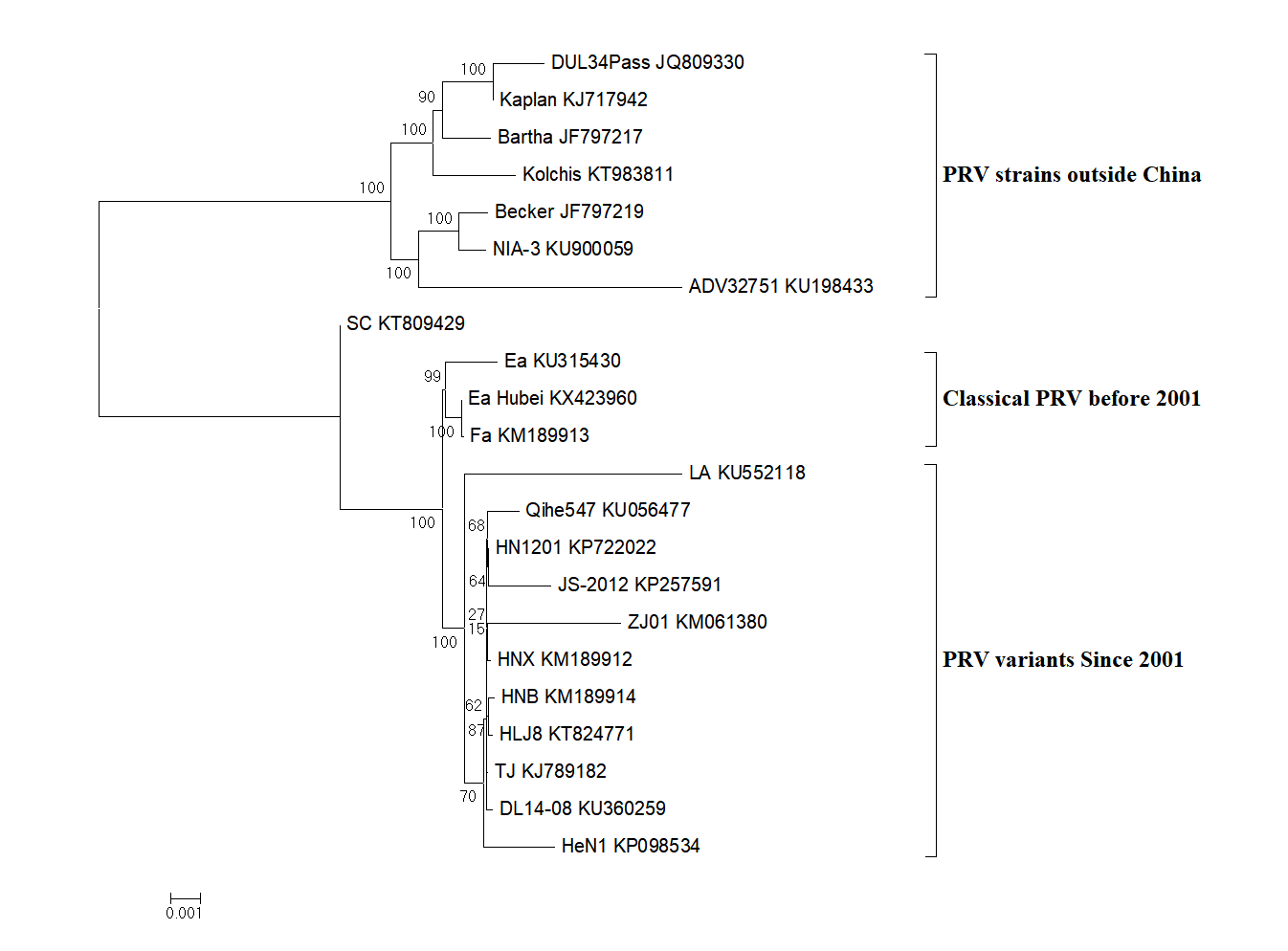

Supplement: S8 Fig — The analysis was performed by using the distance-based neighbor-joining method in MEGA4 software. The Genbank accession numbers are included in the names of all taxons. (TIF) [file ppat.1006777.s009.tif]
